# Supplementary material for: Evaluation of Tacrolimus’ Adverse Effects on Zebrafish in Larval and Adult Stages by Using Multiple Physiological and Behavioral Endpoints
Source: Biology (Basel). 2024 Feb 10;13(2):112. doi: 10.3390/biology13020112 (PMC10886482; doi:10.3390/biology13020112)
Supplement: Supplementary file 1 [file biology-13-00112-s001.zip › biology-2843307-supplementary.pdf]

Supplementary Data

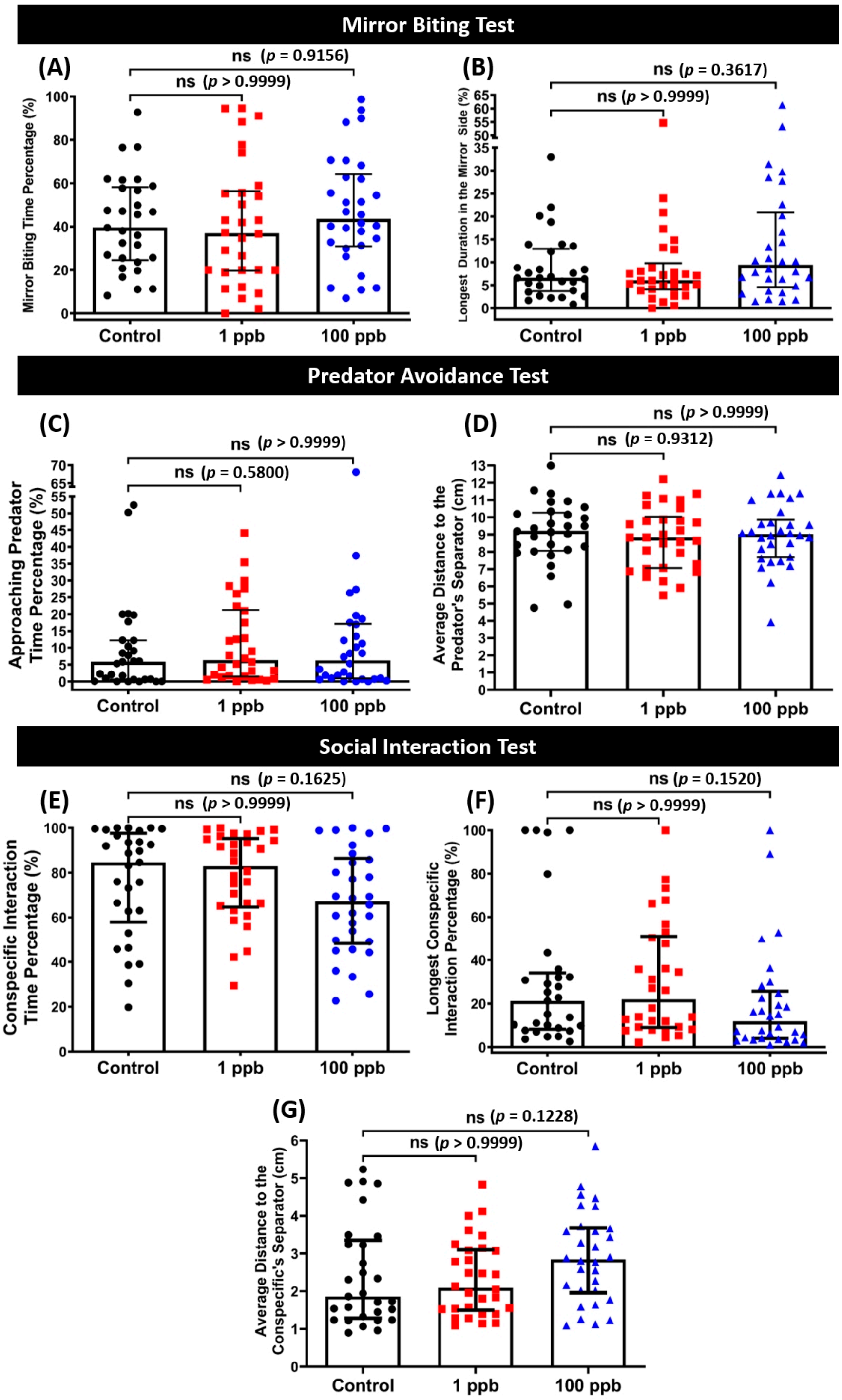

Figure S1. **(A)** Mirror biting time percentage and **(B)** longest duration in the mirror side from the mirror biting test, **(C)** approaching predator time percentage and **(D)** average distance to the predator's separator from predator avoidance test, and **(E)** conspecific interaction time percentage, **(F)** longest conspecific interaction percentage, and **(G)** average distance to the conspecific's separator from the social interaction test of zebrafish after being exposed to tacrolimus at two different concentrations; 1 ppb (red) and 100 ppb (blue) compared to control (black). The data are expressed as the median with interquartile range. The statistical analyses were conducted by Kruskal-Wallis test, which continued with Dunn's multiple comparisons test as a follow-up test (*n* control = 29, *n* 1 and 100 ppb = 30).

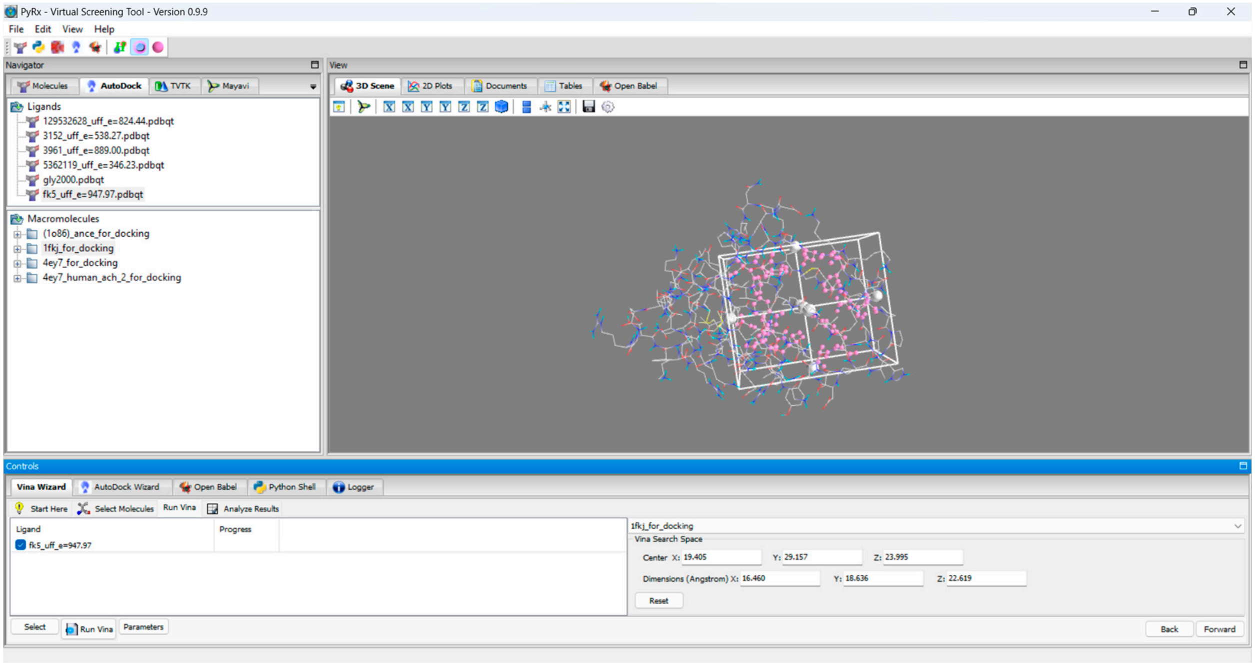

Figure S2. Amino acids observed in the initial extracted complex conformation (complex from the database).

Table S1. Reverse docking results of FKBP12 and tacrolimus (amino acids interaction with tacrolimus).

3D Conformation	Binding Energy (kcal/mol)
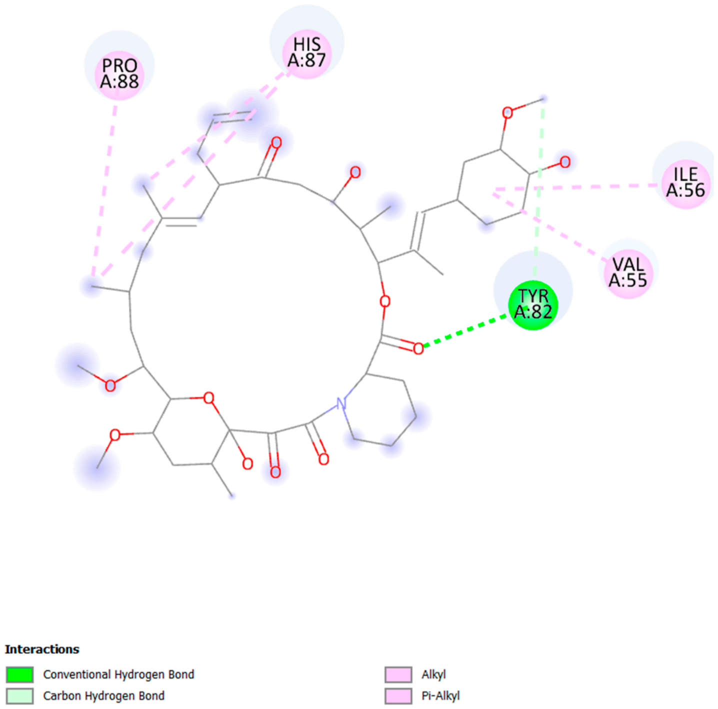	-5.8

<table>
<tr>
<td>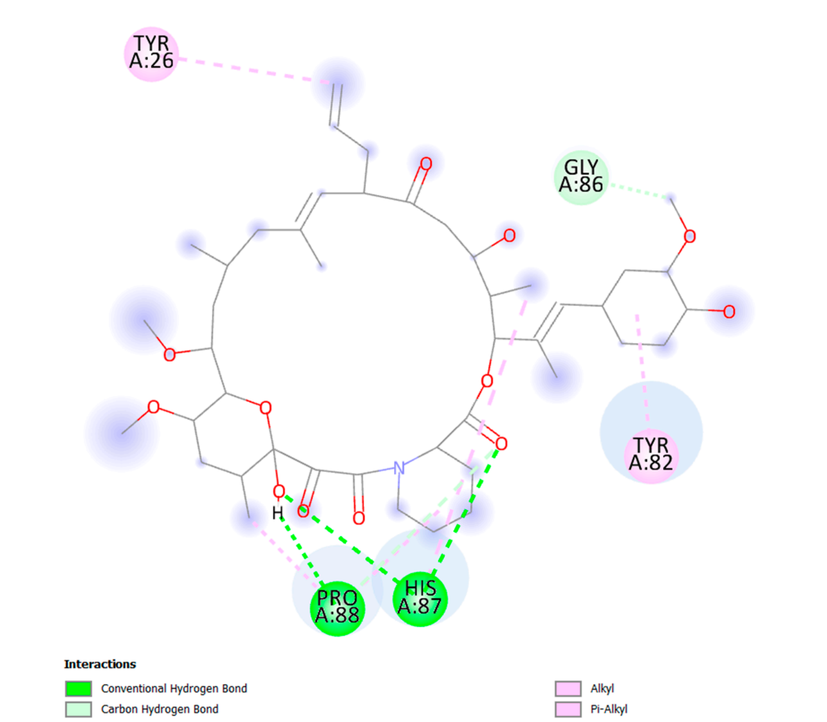
</td>
<td>-5.7</td>
</tr>
<tr>
<td>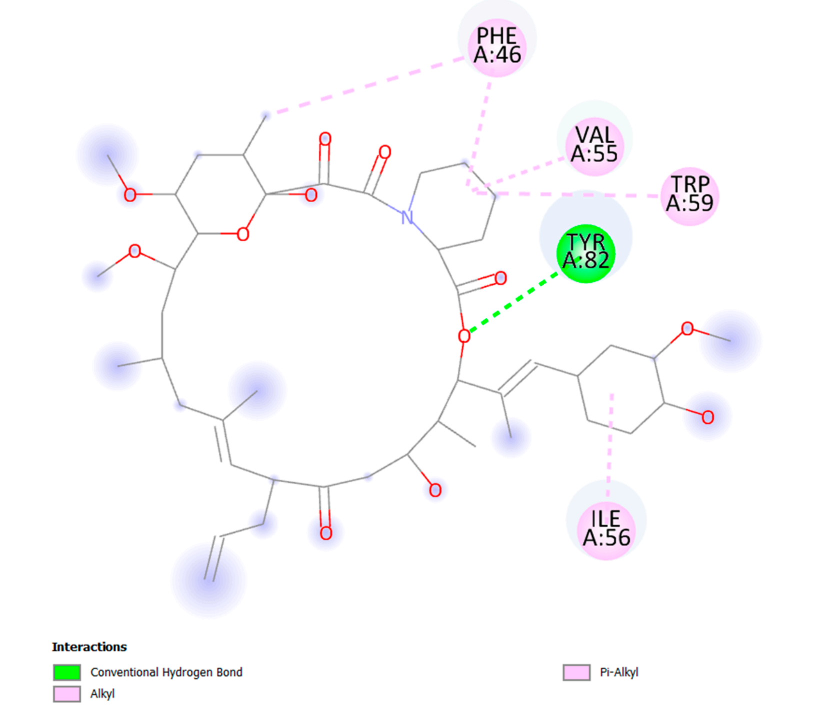
</td>
<td>-5.5</td>
</tr>
</table>

<table>
<tr><td>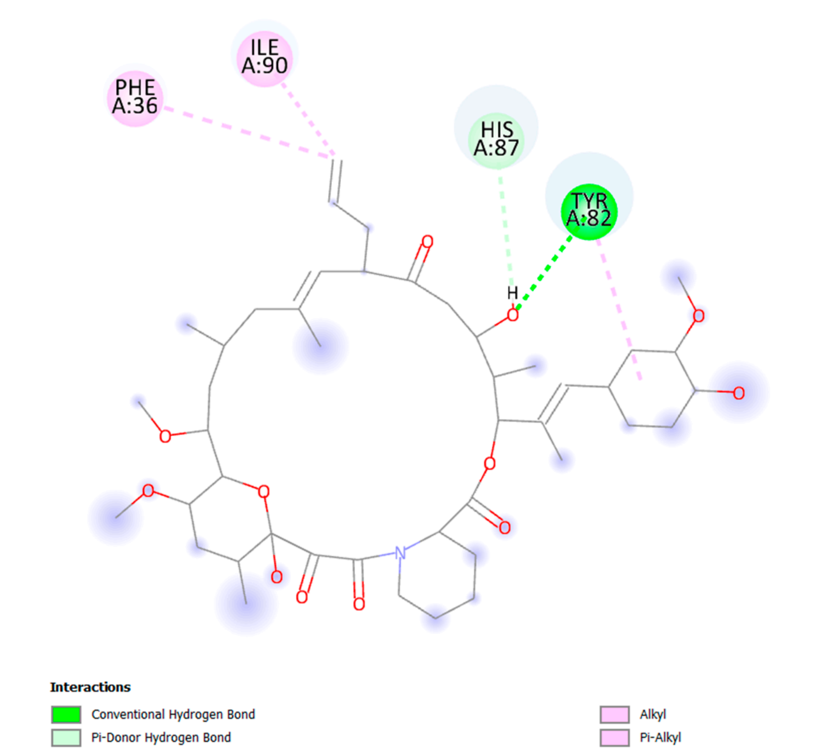
</td><td>-5.5</td></tr>
<tr><td>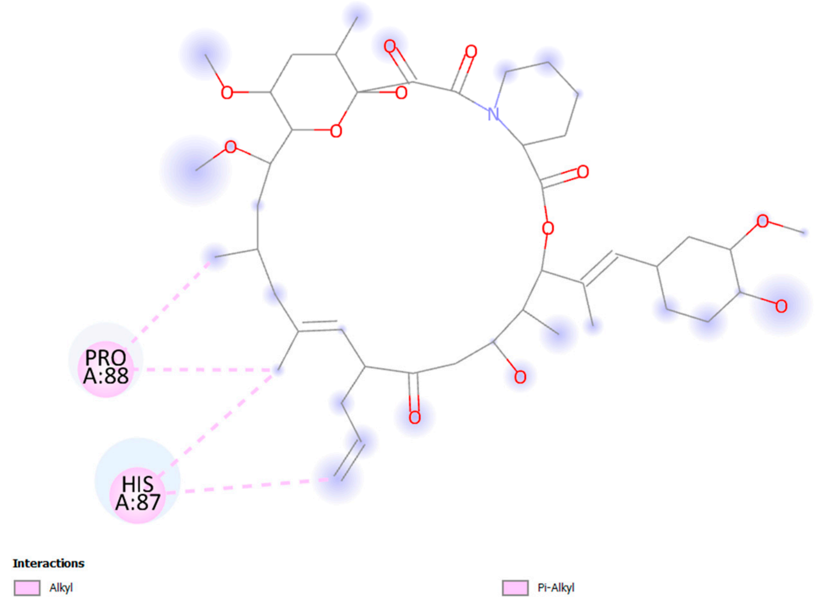
</td><td>-5.4</td></tr>
</table>

<table>
<tr>
<td>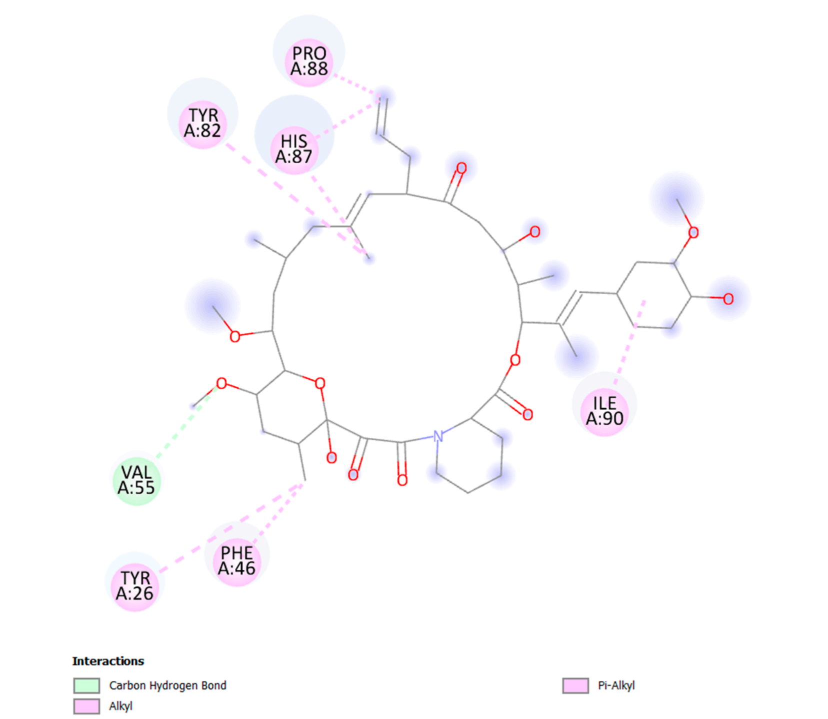
</td>
<td>-5.4</td>
</tr>
<tr>
<td>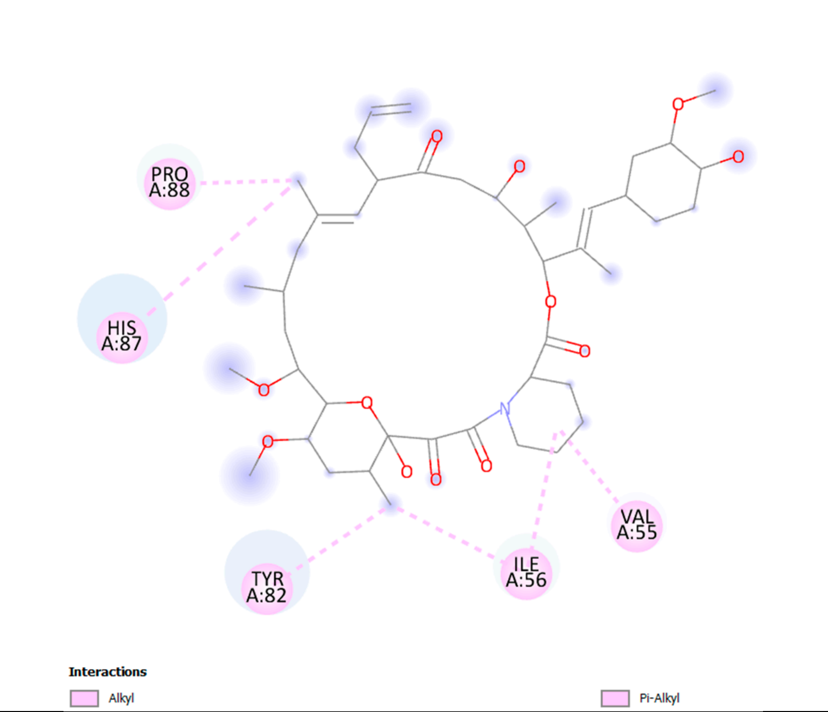
</td>
<td>-4.3</td>
</tr>
</table>

<table>
<tr><td>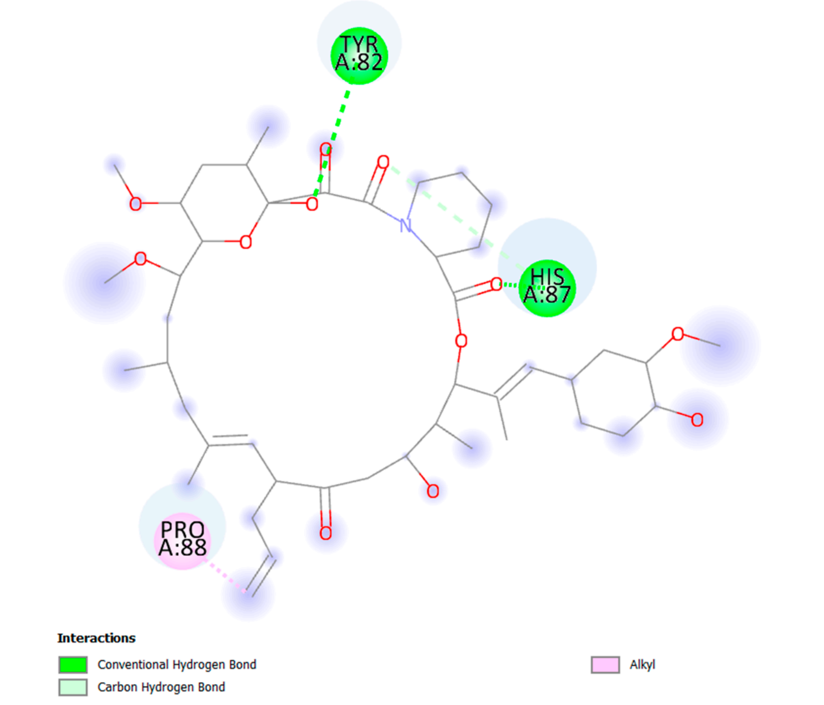
</td><td>-4.1</td></tr>
<tr><td>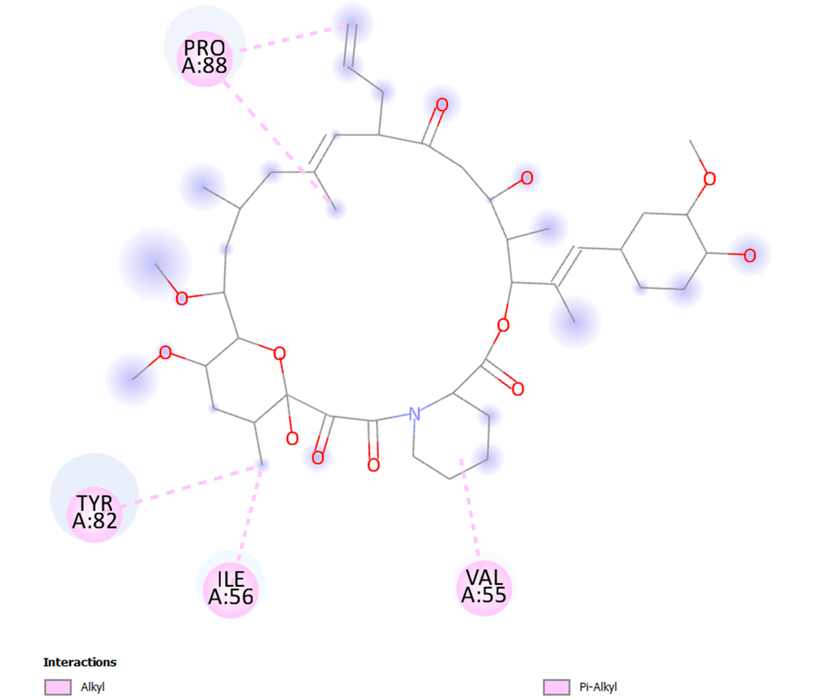
</td><td>-4</td></tr>
</table>

Table S2. Grid size of thing binding site used for the reverse molecular docking procedure.

	X	Y	Z
Center (Å)	19.405	29.157	23.995
Dimension (Å)	16.460	18.636	22.619
